# Comprehensive Design Method of a High-Frequency-Response Fast Tool Servo System Based on a Full-Frequency Error Control Algorithm

**DOI:** 10.3390/mi12111354

**Published:** 2021-10-31

**Authors:** Zelong Li, Chaoliang Guan, Yifan Dai, Shuai Xue, Lianmin Yin

**Affiliations:** 1 College of Intelligence Science and Technology, National University of Defense Technology, 109 Deya Road, Changsha 410073, China; lizelong0706@163.com (Z.L.); dyf@nudt.edu.cn (Y.D.); shuaixue1990@163.com (S.X.); lianminy@163.com (L.Y.); 2 Hunan Key Laboratory of Ultra-Precision Machining Technology, Changsha 410073, China; 3 Laboratory of Science and Technology on Integrated Logistics Support, National University of Defense Technology, 109 Deya Road, Changsha 410073, China

**Keywords:** fast tool servo, piezoelectric actuator, Prandtl–Ishlinskii hysteresis model, feedforward compensator, zero phase error control

## Abstract

With the development of optoelectronic information technology, high-performance optical systems require an increasingly higher surface accuracy of optical mirrors. The fast tool servo (FTS) based on the piezoelectric actuator is widely used in the compensation machining of high-precision optical mirrors. However, with the low natural frequency of mechanical structures, hysteresis of the piezoelectric actuators, and phase delay of the control systems, conventional FTS systems face problems such as a low working frequency and a large tracking error. This study presents a method for the design of a high-performance FTS system. First, a flexure hinge servo turret with a high natural frequency was designed through multi-objective optimization and finite element simulations. Subsequently, a composite control algorithm was proposed, targeting the problems of hysteresis and phase delay. The modified Prandtl–Ishlinskii inverse hysteresis model was used to overcome the hysteresis effect and a zero-phase error tracker was designed to reduce the phase error. The experimental results reveal that the tracking error of the designed FTS system was <10% in the full frequency range (0–1000 Hz).

## 1. Introduction

Ultra-precision optical elements have been widely used in modern optical systems, such as imaging systems, early warning and detection systems, and high-power lasers, and their accuracy typically needs to be >0.1 μm. Ultra-precision lathes can realize the one-time formation of lightweight ultra-precision aluminum mirrors by multi-axis linkage, which allows for rapid manufacturing in batches. Because imaging optical systems develop from infrared to visible light and then to short wavelengths, a higher machining accuracy is required in optical mirrors. With factors such as clamping and cutting errors in the machining process of ultra-precision lathes [1], achieving the accuracy requirements for optical mirrors through one-time machining is difficult [2]. Therefore, scholars in China and abroad have proposed the concept of error compensation machining with fast servo tools. The real-time compensation of machining errors was shown to improve the machining accuracy of optical mirrors [3,4]. Due to the effects of various machining factors, the machining error surface shape is complex and distributed at various frequencies [5]. Limited by the performance of machining tools and the accuracy of error measurements, the existing compensation machining methods mainly target low-frequency surface errors and cannot handle medium- and high-frequency surface errors. To meet the precision requirements of ultra-precision optical mirrors suitable for high-performance optical systems, the transition from low-frequency surface compensation machining to full-frequency surface compensation machining is critical. The development of fast servo tools with a small tracking error in the complete frequency band (0–1000 Hz) is the key technical problem in achieving this transition. Considering the error frequency, complex surface shape, and machining efficiency, it is essential to develop fast servo tools that can adapt to complex morphology machining with a tracking error of <10% in the full frequency range (0–1000 Hz).

The fast servo tool based on the piezoelectric actuator has motion performance with a high-frequency response and high resolution, and it has been widely used in the field of ultra-precision turning. However, its application is limited by the low natural frequency of the mechanical structure, the hysteresis effect of the piezoelectric actuator, and the phase delay problems and amplitude attenuation that occur during high-frequency motion. The conventional proportion integration differentiation (PID) control algorithm cannot be easily used to overcome the nonlinear effect and phase delay of a piezoelectric actuator [6]. Therefore, researchers have tried various advanced control theories to improve the control performance of fast servo tools [7,8]. Zhu et al. improved the mechanical structure of the fast servo tool and optimized the PID controller and dynamic inversion-based feedforward compensation by using the Nyquist diagram. With this design, a maximum closed-loop bandwidth of 1730 Hz was achieved, and the tracking error at 100 Hz was less than ±1.5% [9]. Wu et al. proposed a composite control strategy composed of the online sequential extreme learning machine feedforward model and the PID feedback controller. Under a closed-loop bandwidth of 200 Hz, the linearity was >0.54% [10]. Zhou et al. proposed an improved adaptive feedforward cancellation method for the trajectory tracking of fast tool servo (FTS) by fractional calculus; a tracking error of 1.63% at 200 Hz was obtained [11].

Piezoelectric actuators are widely used in various fields, such as atomic force microscopy [12] and nano-optics [13]. In addition, researchers have attempted to eliminate the hysteresis effect of a piezoelectric actuator by establishing various hysteresis models [14,15]. Fang et al. proposed the Bouc–Wen model and identified the model parameters with the modified particle swarm optimization algorithm. Combined with the closed-loop control algorithm, a tracking error of 2.9% at 10 Hz was obtained [16]. Hu et al. proposed a convolutional neural network model based on the Prandtl–Ishlinskii model. The standard error of the proposed hysteresis model in predicting the displacement at unmodelled frequencies was reduced by 18.74–36.75% [17]. Currently, the servo control algorithms for fast servo tools and piezoelectric actuators can effectively address the hysteresis phenomenon of a piezoelectric actuator. However, the algorithms are mostly applied in the low-frequency range, and few studies have focused on the tracking performance of fast servo tools in the medium- and high-frequency ranges. Overall, it is critical and challenging to develop FTS systems with a full-frequency range tracking error of <10%.

In this study, considering the mechanical structure, hysteresis effect, and phase delay, a method for the design of an FTS system with a full-frequency (0–1000 Hz) tracking error of <10% was proposed. Through multi-objective optimization, a flexure hinge tool holder with a high natural frequency was designed. Furthermore, for the hysteresis of the piezoelectric actuator and high-frequency phase delay, a composite control algorithm was proposed. The simulation experiment showed that the designed FTS system had a tracking error of <10% in the full frequency band (0–1000 Hz), which laid the foundation for high-precision error compensation machining and complex surface cutting.

## 2. Comprehensive FTS System Design Method Based on Structure Design and Full-Frequency Error Control

**Figure 1:**
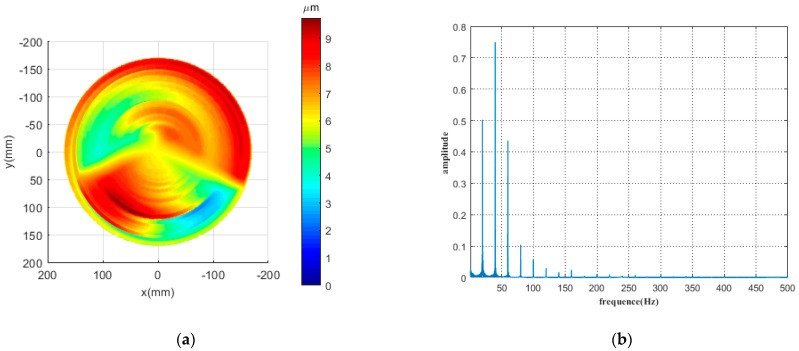
(**a**) A typical error profile; (**b**) spectrogram of machining trajectory.

Figure 1a shows a typical error profile [18]. According to the actual processing speed and feed rate, it is converted into the processing track of the fast servo tool, and then the processing track is analyzed by a frequency spectrum. As shown in Figure 1b, the errors at high, middle, and low frequencies are distributed across various frequency bands.

Therefore, in order to improve the accuracy of the compensation processing, the FTS system needs to maintain a small tracking error in each frequency band in high, middle, and low frequencies. Considering the adaptability of the compensation processing method to different complex surface errors, the compensation processing efficiency, and the economy and complexity of the FTS system, it is relatively easy to extend the frequency band error control range to 0–1000 Hz and control the tracking error amplitude within 10%. For this reason, it is necessary to thoroughly study the mechanical structure, hysteresis effect, and phase delay of the FTS system. A flexure hinge tool holder with a high natural frequency was first designed to meet the basic conditions of high-frequency-response FTS motion. Next, a composite control algorithm was proposed to solve the issues of hysteresis and phase delay in the FTS system, allowing the tracking error to be less than 10% for each frequency band.

### 2.1. Design of the Flexure Hinge Tool Holder with a High Natural Frequency

In an FTS system, the flexure hinge tool holder transmits the output displacement of the piezoelectric actuator. The output displacement of the tool holder driven by the piezoelectric actuator can be approximated as the displacement of the tool. To ensure the high-frequency-response motion performance of the fast servo tool, the natural frequency of the flexure hinge tool holder should be >1000 Hz. The greater the stiffness of the flexure hinge is, the higher the natural frequency will be. However, the stiffness of the flexure hinge may lead to the displacement loss of the piezoelectric actuator. A greater stiffness corresponds to a smaller maximum output displacement, as indicated by Equation (1):

(1) $$\Delta L=\Delta L_{0}(\frac{k_{p}}{k_{p}+k})$$

where $\Delta L_{0}$ is the maximum output displacement of the piezoelectric actuator when it is under no load, ΔL is the actual maximum output displacement of the FTS system, $k_{p}$ is the stiffness of the piezoelectric actuator, and k is the stiffness of the flexure hinge.

Considering the displacement loss and natural frequency of the FTS system, a multi-objective optimization method was used to optimize the tool holder structure. First, a flexure hinge with a low stiffness was designed, reducing the displacement loss to the maximum possible extent. Subsequently, the mass of the flexure hinge was reduced to the highest extent using structure optimization methods, such as adopting a hollow design, to enhance the natural frequency of the system.

**Figure 2:**
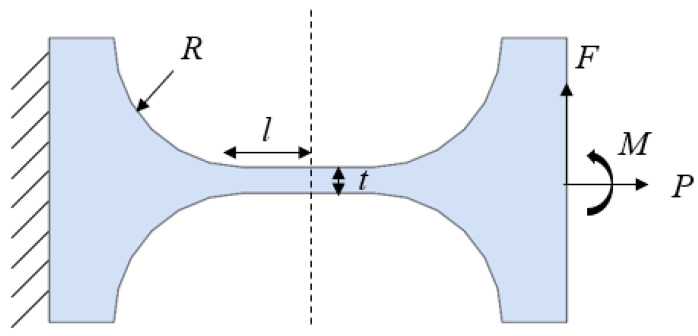
Stress diagram of the straight-beam flexure hinge.

This study adopted the structure of a straight-beam hinge, as shown in Figure 2. *R* is the arc radius of the hinge, *l* is the length of the straight beam, *t* is the minimum thickness of the straight beam, *F* is the driving force of the hinge, and *M* is the bending moment on the hinge. The overall rotational stiffness of the straight-beam hinge is calculated using Equation (2) [19]:

(2) $$\begin{array}{l} k=\frac{Ebt^{3}}{12l}+\frac{EbR^{2}}{12f}\\ f=\frac{12s^{4}(2s+1)}{{\left(4s+1\right)}^{5/2}}\arctan\sqrt{4s+1}+\frac{2s^{3}(6s^{2}+4s+1)}{{\left(4s+1\right)}^{2}(2s+1)} \end{array}$$

where *E* is the elastic modulus of the material, *s* = *R*/*t*, and the rotational stiffness, *k*, has a unit of N·μm/rad.

**Figure 3:**
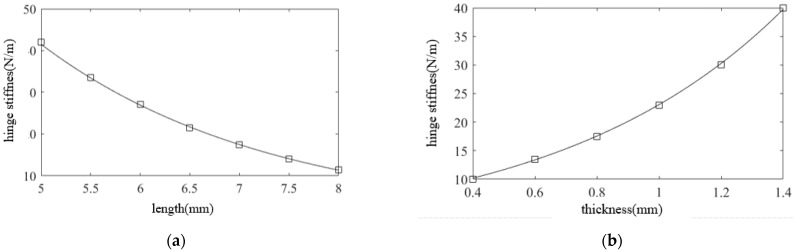
(**a**) Straight beam length; (**b**) minimum thickness.

**Figure 4:**
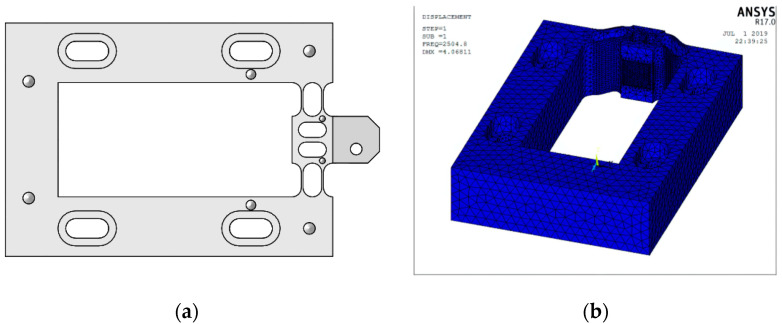
(**a**) Model of the flexure hinge tool holder; (**b**) first-order vibration mode.

As displayed in Figure 3, the longer the straight beam is, the lower the hinge stiffness is; meanwhile, the larger the minimum thickness is, the higher the hinge stiffness is. According to the practical machining conditions, the primary dimensions of the hinge were designed as follows: *l* = 7 mm, *t* = 0.6 mm, *R* = 2 mm. The material used was 65 Mn. The stiffness of the designed flexure hinge was 11.6 N/μm. To reduce the mass of the moving part in the device and increase the natural frequency, a hollow structure was adopted in the middle part of the tool holder. Considering the device installation and the tool clamping part, the final model of the designed flexure hinge tool holder is shown in Figure 4a. Assuming that the stiffness of the piezoelectric actuator was 200 N/μm and the output displacement was 10 μm, the displacement loss was ∆*L* − ∆*L*_0_ = 0.55 μm, according to Equation (1). The natural frequency was determined using the ANSYS software, and the first-order vibration mode of the hinge was obtained (Figure 4b). The first-order natural frequency of the hinge was 2504.8 Hz, which was considerably higher than the maximum working frequency of the system (1000 Hz); thus, an FTS motion frequency of >1000 Hz could be obtained.

### 2.2. Composite Control Algorithm

**Figure 5:**
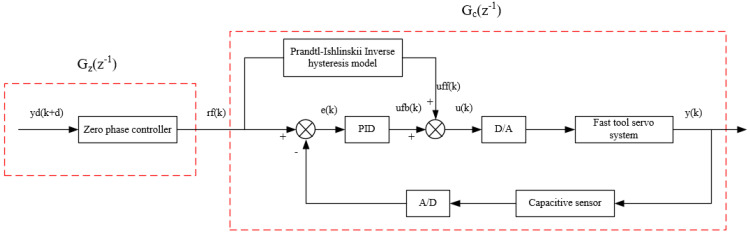
Block diagram of the composite control algorithm.

To address the nonlinear effect of the piezoelectric actuator and the high-frequency phase delay of the FTS system, a composite control algorithm, as shown in Figure 5, was proposed. First, a PID closed-loop control system based on the Prandtl–Ishlinskii inverse hysteresis model was designed to eliminate the hysteresis effect of the piezoelectric actuator. Then, aiming at the system phase delay, a zero-phase error controller, *G_z_*(*z*^−1^), was designed on the basis of the closed-loop system, *G_c_*(*z*^−1^), to reduce the tracking error of the FTS system at high frequencies.

#### 2.2.1. PID Control Based on the Modified Prandtl–Ishlinskii Inverse Hysteresis Model

The PID control based on the Prandtl–Ishlinskii inverse hysteresis model was developed in two steps. First, the Prandtl–Ishlinskii inverse hysteresis model was established. This model was then combined with the PID control algorithm to eliminate the hysteresis effect of the piezoelectric actuator.

In the FTS system, the stiffness of the flexure hinge demonstrates a certain nonlinear effect, leading to a nonideal symmetrical distribution in the hysteresis curve of the piezoelectric actuator [20]. The traditional hysteresis curve can only describe the case of symmetrical distribution [21]. Equation (3) describes the traditional Prandtl–Ishlinskii hysteresis model. For asymmetric hysteresis curves, an asymmetric term is typically introduced into the Prandtl–Ishlinskii hysteresis model to improve the identification accuracy [22].

(3) $$\begin{array}{l} Y(k)=\sum_{i=0}^{N-1}w_{i}y_{ri}[u,y_{i}](t)=\sum_{i=0}^{N-1}w_{i}\max\{u(k)-r_{i},\min\{u(k),y(k-1)\}\}\\ r_{i}=\frac{i}{N}\max\left(\left|u(k)\right|\right),i=0,1,...,N-1 \end{array}$$

where *Y*(*k*) is the output value, *u* is the input value, *r_i_* is the threshold of the Prandtl–Ishlinskii hysteresis model, and *w_i_* is the weight of the model. By comparing the identification results of different nonlinear terms, the modified hysteresis model is expressed in Equation (4):

(4) $$Y(k)=ku^{\frac{1}{3}}+\sum_{i=0}^{N-1}w_{i}y_{ri}[u(k),y_{i}](t)=ku^{\frac{1}{3}}+\sum_{i=0}^{N-1}w_{i}\max\{u(k)-r_{i},\min\{u(k),y(k-1)\}\}$$

where *u*^(1/3)^ is the introduced asymmetric term, and *k* is its coefficient.

**Figure 6:**
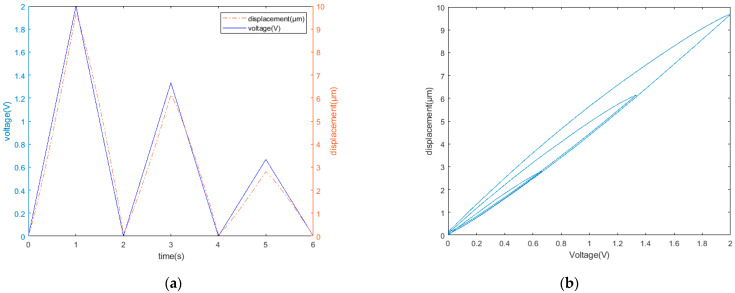

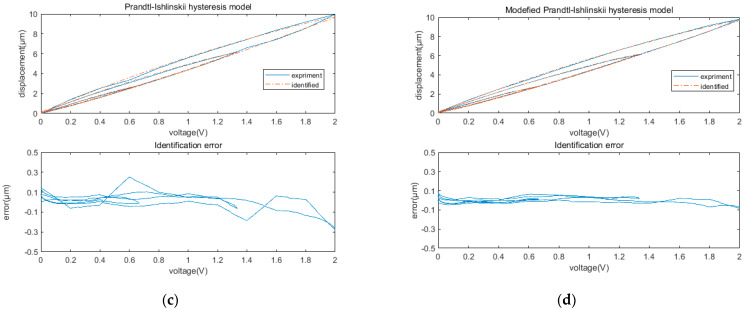
(**a**) Input voltage and output displacement; (**b**) experimentally obtained hysteresis curve. Identification results of the (**c**) traditional Prandtl–Ishlinskii model and (**d**) modified Prandtl–Ishlinskii model.

Using a group of triangular wave signals with different amplitudes, the identification effects of the traditional and modified Prandtl–Ishlinskii hysteresis models were compared, as shown in Figure 6. The adopted identification algorithm was the gradient descent method. The identification error of the traditional Prandtl–Ishlinskii hysteresis model was 6% and that of the modified Prandtl–Ishlinskii hysteresis model was 1.9%, which indicates a significant improvement in the identification accuracy.

**Figure 7:**
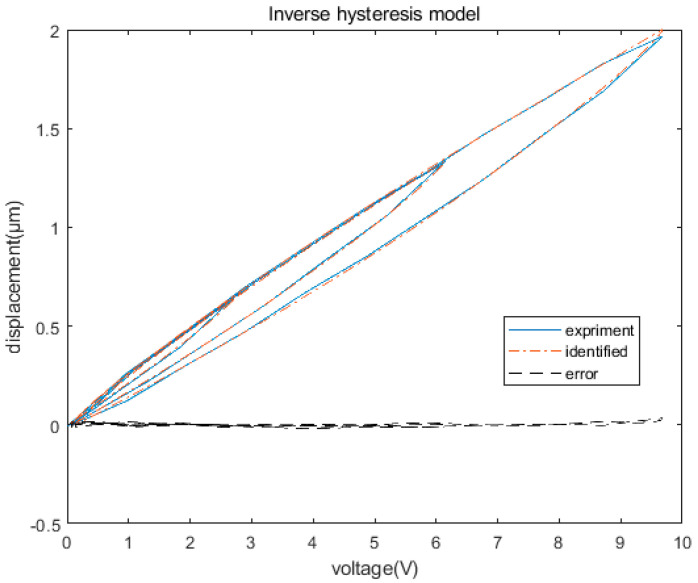
Identification results of the Prandtl–Ishlinskii inverse hysteresis model.

Although the inverse hysteresis model can be derived from the hysteresis model described in Equation (4), the derivation process is highly complex. Because the inverse hysteresis model is also a hysteresis model, it can be directly identified according to the input voltage and output displacement of the piezoelectric actuator [23]. In this study, the direct approach was adopted to identify the inverse hysteresis model. The identification results are shown in Figure 7, demonstrating an identification error of 2.3%, and the identified parameters are listed in Table 1.

**Figure 8:**
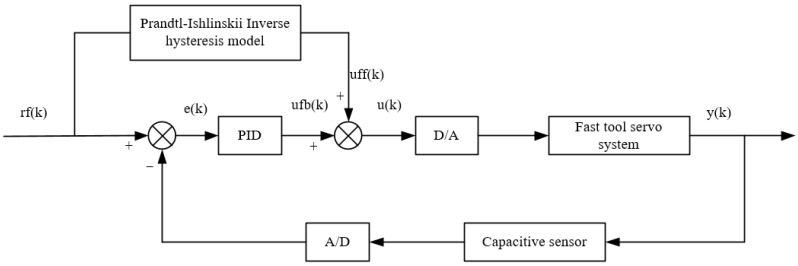
PID control based on the modified Prandtl–Ishlinskii inverse hysteresis model.

The identified Prandtl–Ishlinskii inverse hysteresis model was then connected in series in front of the piezoelectric actuator for feedforward correction and combined with the PID controller for closed-loop control. The hysteresis effect of the piezoelectric actuator was pre-compensated for by the feedforward controller and the error signal was feedback controlled with a PID control. The control block diagram is shown in Figure 8.

First, the reference signal, rf(k), enters the inverse hysteresis model, obtaining the output voltage, uff(k), of the feedforward controller. In the meantime, the capacitive sensor detects the output displacement, y(k), of the FTS system and compares it with the reference signal, r(k), thereby obtaining the error signal, e(k). The error signal, e(k), passes through the PID controller, yielding the feedback control signal ufb(k), which is then superimposed with the feedforward control signal, uff(k), to obtain the final output signal, u(k). This process is presented in Equation (5). Through the PID control method based on the modified Prandtl–Ishlinskii inverse hysteresis model, the hysteresis effect of the piezoelectric actuator can be theoretically eliminated.

(5) $$\begin{array}{l} u(k)=uff(k)+\mathrm{ufb}(k)=Y^{-1}(rf(k))+Kp(k)*e(k)+K_{I}\sum_{i=1}^{k}e(i)-K_{D}[x(k)-x(k-1)]\\ Y^{-1}(rf(k))=ku^{\frac{1}{3}}+\sum_{i=0}^{N-1}w_{i}y_{ri}[rf(k),y_{i}](t)=ku^{\frac{1}{3}}+\sum_{i=0}^{N-1}w_{i}\max\{rf(k)-r_{i},\min\{rf(k),y(k-1)\}\} \end{array}$$

#### 2.2.2. Zero Phase Error Controller

**Figure 9:**
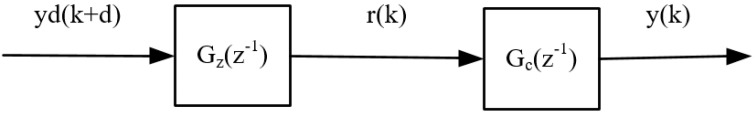
Block diagram of zero-phase error tracking control.

To eliminate the phase delay of the FTS system, a zero-phase error controller, *G_z_*(*z*^−1^), was used to correct the phase of the closed-loop control system, *G_c_*(*z*^−1^), as displayed in Figure 9. By establishing the Prandtl–Ishlinskii inverse hysteresis model, the closed-loop control system, *G_c_*(*z*^−1^), could essentially eliminate the nonlinear effect of the piezoelectric actuator. Therefore, the closed-loop system could be considered a linear model, and the controlled closed-loop control system, *G_c_*(*z*^−1^), could be described using Equation (6):

(6) $$G_{c}(z^{-1})=\frac{z^{-d}B_{c}(z^{-1})}{A_{c}(z^{-1})}=\frac{z^{-d}B_{c}{}^{a}(z^{-1})B_{c}{}^{u}(z^{-1})}{A_{c}(z^{-1})}$$

where *z^−d^* is the *d*th-order delay caused by the closed-loop system, *B_c_*(*z*^−1^) and *A_c_*(*z*^−1^) are expressed by Equation (7), *B_a_*(*z*^−1^) is the zero in the unit circle of the closed-loop system, and *B_c_^u^*(*z*^−1^) is the zero outside the unit circle of the closed-loop system.

(7) $$\begin{array}{l} B_{c}(z^{-1})=b_{c0}+b_{c1}z^{-1}+\ldots +b_{cm}z^{-m},b_{c0}\neq 0\\ A_{C}(z^{-1})=1+a_{c1}z^{-1}+\ldots +a_{cn}z^{-n} \end{array}$$

Because the zero outside the unit circle of the closed-loop system cannot be eliminated directly, the zero-phase error controller designed for the closed-loop system can be expressed as [24,25]:

(8) $$G_{z}(z^{-1})=\frac{z^{d+s}A_{c}(z^{-1})B_{c}{}^{u*}(z^{-1})}{B_{c}{}^{a}(z^{-1}){\left[B_{c}{}^{u}(1)\right]}^{2}}$$

where *s* is the number of zeros outside the unit circle of the closed-loop system and B_c_^u^*(*z*^−1^) is the conjugate of *B_c_^u^*(*z*^−1^), as presented in Equation (9):

(9) $$\begin{array}{l} B_{c}{}^{u}(z^{-1})=b_{c0}+b_{c1}z^{-1}+\ldots +b_{cs}z^{-s}\\ B_{c}{}^{u*}(z^{-1})=b_{cs}+b_{cs-1}z^{-1}+\ldots +b_{c1}z^{-s} \end{array}$$

When there are no poles outside the unit circle in the closed-loop system, Equation (8) can be transformed into:

(10) $$G_{z}(z^{-1})=\frac{z^{d}A_{c}(z^{-1})}{B(z^{-1})}$$

**Figure 10:**
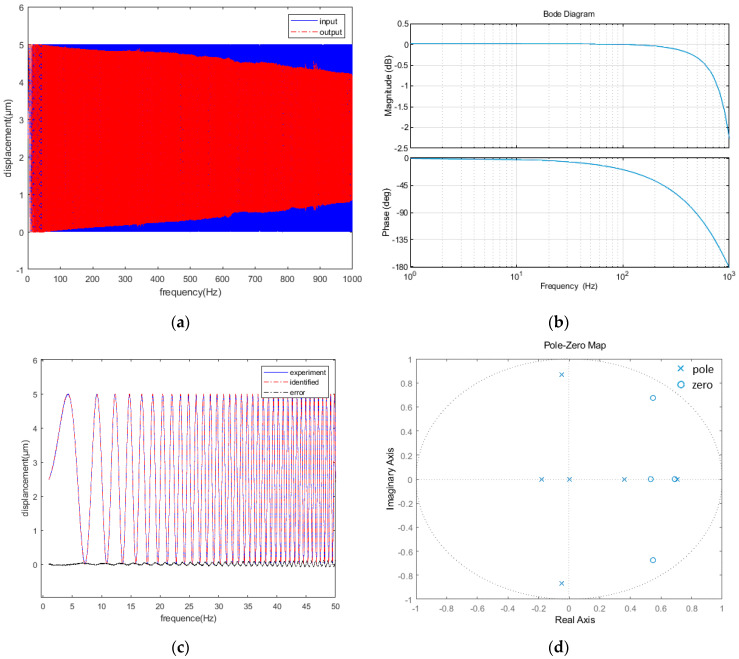
(**a**) Sweep signal at 0–1000 Hz frequency; (**b**) Bode diagram of the closed-loop system; (**c**) identification results; (**d**) zero-pole distribution.

The basic steps of designing the zero-phase error controller are as follows. (a) The closed-loop system, *G_c_*(*z*^−1^), was identified, and its zero-pole distribution was obtained. (b) An appropriate zero-phase error controller, *G_z_*(*z*^−1^), was designed according to the zero-pole distribution of the closed-loop system. After the completion of the design based on the Prandtl–Ishlinskii inverse hysteresis model in Section 3, the 0–1000 Hz transfer function of the closed-loop system was identified using the system identification toolbox of MATLAB. Then, the zero-pole distribution of the closed-loop system was obtained. Figure 10a presents the result of a sinusoidal frequency sweep at 0–1000 Hz and Figure 10b shows the Bode diagram of the closed-loop system. The phase delay and amplitude attenuation of the system increased gradually with the increase in the frequency. Figure 10c shows the identification results of the closed-loop system, *G_c_*(*z*^−1^), with an identification degree of 96.5%. Equation (11) presents the identified *G_c_*(*z*^−1^).

(11) $$G_{c}(z^{-1})=\frac{4.154-9.631z^{-1}+10.23z^{-2}-5.511z^{-3}+1.154z^{-4}}{1-0.7852z^{-1}+0.7299z^{-2}-0.614z^{-3}+0.05156z^{-4}+0.03447z^{-5}}z^{-5}$$

Figure 10d displays the zero-pole distribution of the closed-loop system, showing no zeros outside the unit circle. According to Equations (8) and (10), the zero-phase controller can be described using Equation (12). With the design of the zero-phase error controller, the high-frequency phase delay of the FTS system could be theoretically eliminated.

(12) $$G_{z}(z^{-1})=\frac{0.2299-0.1805z^{-1}+0.1666z^{-2}-0.1411z^{-3}+0.01185z^{-4}+0.007925z^{-5}}{1-2.293z^{-1}+2.495z^{-2}-1.367z^{-3}+0.3167z^{-4}}$$

## 3. Experimental Results and Discussions

### 3.1. Experimental Results

**Figure 11:**
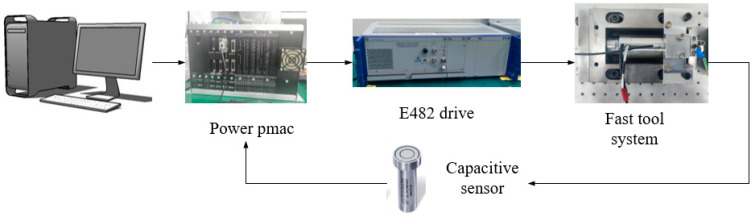
Experimental setup.

The experimental setup is shown in Figure 11; it consisted of a piezoelectric actuator (PI225.40), a power amplifier (E482.00), a Power PMAC motion controller, a capacitive sensor (D-510.051), and a flexure hinge tool holder. The piezoelectric actuator transmitted the displacement through the flexure hinge tool holder. The input voltage of the E482 power amplifier was 0–10 V and the magnification was 10. The maximum servo frequency of the motion controller was 100 kHz. The output bandwidth of the capacitive sensor and power amplifier was >1 kHz.

For clarity, PID + HC is used to represent PID plus the hysteresis compensation control and PID + HC + ZPEC is used to represent PID with the hysteresis compensator and zero-phase error control.

**Figure 12:**
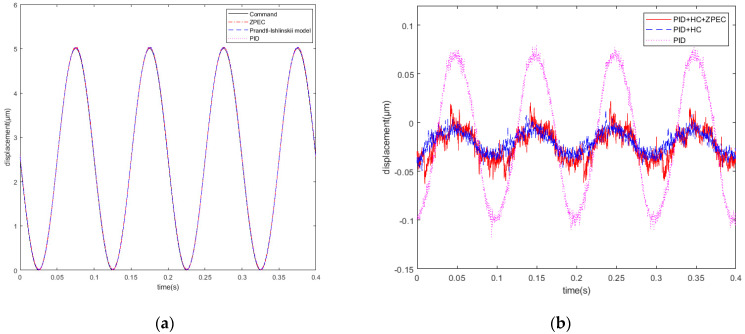
(**a**) Sinusoidal signal at 10 Hz; (**b**) tracking errors of the 10 Hz sinusoidal signal.

As shown in Figure 12, for the 5 μm and 10 Hz sinusoidal signal, the tracking errors of the PID control, PID + HC control, and PID + HC + ZPEC control are 0.1975, 0.0641, and 0.0849 μm, respectively. The experimental results show that the tracking error of PID + HC control decreases by 70% compared with that of the PID control, indicating that the nonlinear effect of the piezoelectric actuator is effectively addressed by introducing the inverse hysteresis model. At 10 Hz, the system phase delay error does not contribute majorly; thus, the tracking error of PID + HC + ZPEC control is close to that of the PID + HC control.

**Figure 13:**
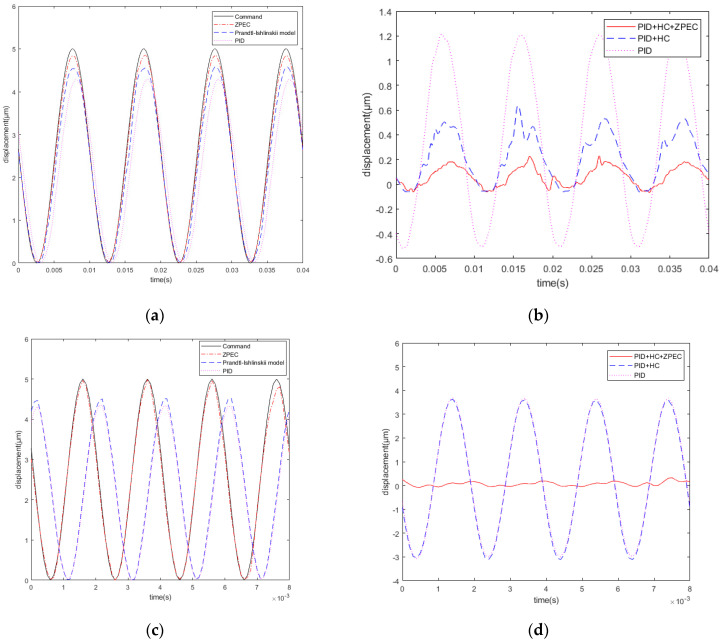
(**a**) Sinusoidal signal at 100 Hz; (**b**) tracking errors of the 100 Hz sinusoidal signal; (**c**) 500 Hz sinusoidal signal; (**d**) tracking errors of the 500 Hz sinusoidal signal.

The tracking results in the case of the 5 μm and 100 Hz sinusoidal signal are shown in Figure 13a,b. The tracking errors are 1.714, 0.6934, and 0.2968 μm for PID control, PID + HC control, and PID + HC + ZPEC control, respectively. Figure 13c,d show the tracking results of the 5 μm and 500 Hz sinusoidal signal; the tracking errors are 6.922, 6.784, and 0.3926 μm for PID control, PID + HC control, and PID + HC + ZPEC control, respectively. The experimental results indicate that the tracking errors of PID and PID + HC increase gradually with the increase in the frequency. After the introduction of the zero-phase controller, the system tracking error decreases considerably. Compared with that of the PID + HC control, the tracking error of PID + HC + ZPEC decreases by 57% at 100 Hz and by 94% at 500 Hz, indicating that PID + HC + ZPEC can effectively overcome the tracking error caused by the system phase delay during high-frequency motion.

**Figure 14:**
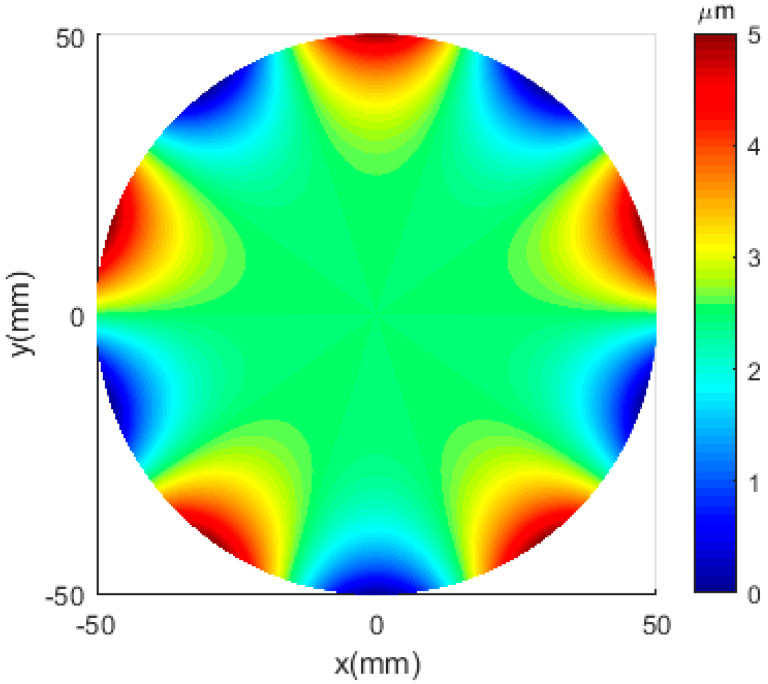
Five-leaved error surface shape.

**Figure 15:**
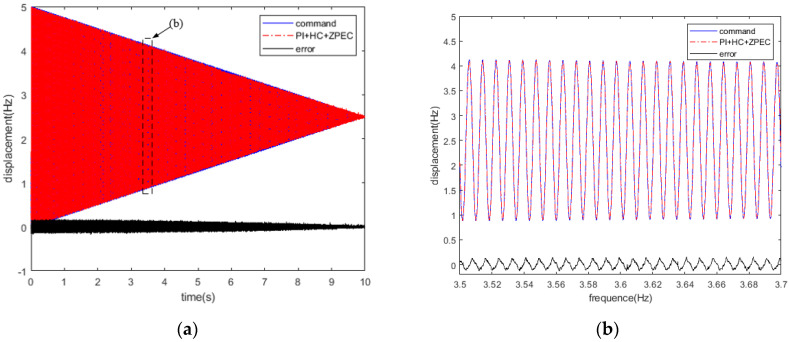
Tracking error of the five-leaved signal; (**a**) five-leaved signal; (**b**) tracking error.

To further verify the control performance of the PID + HC + ZPEC controller, a five-leaved error signal was used in a 0–1000 Hz frequency sweep test. Figure 14 illustrates a typical five-leaved error surface shape. The revolving speed was set to *N* = 1200 r/min; the feed speed was *v* = 2 mm/min; the machining radius was 50 mm. The motion trajectory of the FTS is expressed using Equation (13), and the tracking result is shown in Figure 15. A maximum tracking error of <0.3 μm was obtained.

(13) z(t)=(50−10t)sin(200πt)

**Figure 16:**
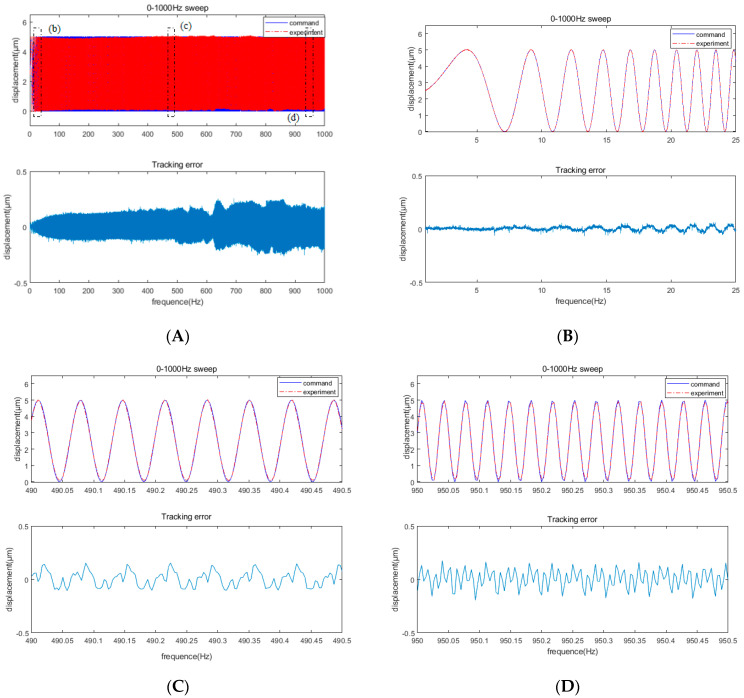
(**A**) Tracking error of the 0–1000 Hz sinusoidal sweep signal; (**B**) local amplification of Region B; (**C**) local amplification of Region C; (**D**) local amplification of Region D.

The test results of a group of 0–1000 Hz sweep signals are presented in Figure 16. Figure 16a displays the tracking result from 0 to 1000 Hz, showing a full-band tracking error of <10%. Figure 16b–d present the local enlarged views of the corresponding regions. Without considering other factors, the fast servo tool can be used to realize error compensation machining at 0–1000 Hz with a tracking error of <10%.

### 3.2. Discussion

Compared with conventional PID algorithms and advanced algorithms, such as the automatic anti-disturbance algorithm, the proposed composite algorithm can effectively overcome the hysteresis effect of a piezoelectric actuator and the high-frequency phase delay in the FTS system. However, it requires the identification of an accurate system model. In practice, the model may vary with the changing machining environment, resulting in the poor correction of the zero-phase controller. Under this circumstance, the model must be re-identified. To improve the robustness of the algorithm, the repetitive controller could be introduced to eliminate the effect of external interferences on the zero-phase controller. In addition, we have previously used the zero-phase controller directly on the PID controller without the Prandtl–Ishlinskii hysteresis compensator. However, because only the PID control could not overcome the nonlinear effect of the piezoelectric actuator, the system identification error was large, which made it difficult to design an accurate zero-phase controller.

## 4. Conclusions

In response to the hysteresis effect of a piezoelectric actuator and the phase delay of the FTS system, a flexure hinge tool holder with a high natural frequency and a composite control algorithm were combined to develop an FTS system with a full-frequency-band (0–1000 Hz) tracking error of <10%. First, through multi-objective optimization and finite element simulations, the flexure hinge tool holder with a natural frequency of >1000 Hz was designed to meet the basic requirements of high-frequency FTS motion. Then, the composite control algorithm was proposed, integrating a PID closed-loop control algorithm based on the modified Prandtl–Ishlinskii inverse hysteresis model and a zero-phase error controller; these addressed the hysteresis effect of the piezoelectric actuator and the phase delay of the FTS system, respectively. The experimental comparison of different control algorithms confirmed that the tracking accuracy of the proposed composite control algorithm substantially improved compared with that of the conventional PID servo control algorithm. The frequency sweep experiment demonstrated that the system had a tracking error of <10% at a working frequency of 0–1000 Hz. Thus, the proposed system can be well adapted to full-frequency high-precision compensation machining and complex surface processing and thus improve the compensation processing accuracy of optical mirrors. The comprehensive method for the design of a high-frequency response fast tool servo system proposed in this paper is comprehensive and universal and can effectively improve the working performance of a fast tool servo system.

## Figures and Tables

**Table 1:** Identified parameters of the Prandtl–Ishlinskii inverse hysteresis model.

i	0	1	2	3	4	5	6	7	8	9
r_i_	0	0.9677	1.9354	2.9031	3.8708	4.8385	5.8062	6.7739	7.7415	8.7092
w_i_	0.29	−0.063	0.0075	−0.0267	−0.0015	−0.0215	0.021	−0.0253	0.0115	−0.047
k	−0.0197									

## Data Availability

The data presented in this study are available on request from the corresponding author. The data are not publicly available because the data are also part of an ongoing study.
